# Phenocave: An Automated, Standalone, and Affordable Phenotyping System for Controlled Growth Conditions

**DOI:** 10.3390/plants10091817

**Published:** 2021-08-31

**Authors:** Fernanda Leiva, Pernilla Vallenback, Tobias Ekblad, Eva Johansson, Aakash Chawade

**Affiliations:** 1 Department of Plant Breeding, Swedish University of Agricultural Sciences, SE-23422 Lomma, Sweden; fernanda.leiva@slu.se (F.L.); eva.johansson@slu.se (E.J.); 2 Lantmännen Lantbruk, SE-26881 Svalöv, Sweden; Pernilla.vallenback@lantmannen.com; 3 MariboHilleshög Research AB, SE-26191 Landskrona, Sweden; tobek79@gmail.com

**Keywords:** affordable, phenotyping, drought, image analysis, automated

## Abstract

Controlled plant growth facilities provide the possibility to alter climate conditions affecting plant growth, such as humidity, temperature, and light, allowing a better understanding of plant responses to abiotic and biotic stresses. A bottleneck, however, is measuring various aspects of plant growth regularly and non-destructively. Although several high-throughput phenotyping facilities have been built worldwide, further development is required for smaller custom-made affordable systems for specific needs. Hence, the main objective of this study was to develop an affordable, standalone and automated phenotyping system called “Phenocave” for controlled growth facilities. The system can be equipped with consumer-grade digital cameras and multispectral cameras for imaging from the top view. The cameras are mounted on a gantry with two linear actuators enabling XY motion, thereby enabling imaging of the entire area of Phenocave. A blueprint for constructing such a system is presented and is evaluated with two case studies using wheat and sugar beet as model plants. The wheat plants were treated with different irrigation regimes or high nitrogen application at different developmental stages affecting their biomass accumulation and growth rate. A significant correlation was observed between conventional measurements and digital biomass at different time points. Post-harvest analysis of grain protein content and composition corresponded well with those of previous studies. The results from the sugar beet study revealed that seed treatment(s) before germination influences germination rates. Phenocave enables automated phenotyping of plants under controlled conditions, and the protocols and results from this study will allow others to build similar systems with dimensions suitable for their custom needs.

## 1. Introduction

One of the most important factors in functional plant biology and growth analysis is plant biomass [1,2]. This parameter is the basis to obtain the net primary production and growth rate in every crop at different growth stages [3,4]. Conventional methods for estimating plant biomass, however, require destructive harvests and are labor-intensive and expensive. Thus, conventional methods for variety evaluation are often based on the final yield for replicated plots under different environments [5]. Since conventional methods to measure plant biomass are destructive, it is challenging to assess the process of development of individual plants at different growth stages. In addition, certain measurements are generally obtained from individual plants that are randomly selected from a plot. Therefore, there is a need to develop more efficient, precise, and accurate methods to assess key agronomical traits for crop monitoring [6]. Another type of seed study is “seed priming and coating”, a pre-sowing technology to treat seeds with one or various agents before germination [7]. These technologies help plants prepare their defense metabolism against certain stress factors [8,9]. Thus, coating and priming can improve seed performance, resulting in faster and better germination and improving plant growth [10,11,12]. Conventional methods and X-ray analysis have been used to measure the growth rate in sugar beet. However, a reliable, quick, and automated method to estimate the early growth of seeds treated with different treatments would make it easier to evaluate new treatments more efficiently.

The current emergence of imaging techniques and recent advances in technology have contributed to further advances in plant phenotyping for the field and controlled conditions. These new image-based systems evaluate genotype-environment interaction through tracking plant growth and health performance in a non-destructive, automated, and high-throughput way [13]. Another advantage of such systems is that they allow the evaluation of a large number of individuals over time, raising the possibility of identifying traits that cannot be tracked by conventional measurements [14] and reducing crop production losses [15,16].

Plant phenotyping systems designed to analyze projected leaf area or canopy biomass over plant development time using a single camera in the visible light range (RGB) have proven to be helpful for the estimation of growth rate, health status, drought or salinity stress, and early vigor [17,18,19,20,21]. Other systems use more sophisticated commercial optical sensors like hyperspectral or multispectral imaging [22]. Hundreds of images can be taken within a short period and have proven to provide reliable information related to foliar and moisture nutrient content, plant health, water content composition parameters for seeds, and leaf area index, applying different vegetation indexes [3,15,23]. Other sensors, such as thermal and chlorophyll fluorescence, can also be integrated to detect abiotic and biotic stresses and photosynthetic performance [24,25,26,27]. The choice of optical sensors mainly depends on the phenotypic variation of interest and image acquisition conditions [26].

Some examples of high-throughput plant phenotyping platforms are the “WIWAM” platforms in Belgium [28], which are systems based on non-invasive automated imaging and precise irrigation of plants. Another similar system is Plant PhenoLab in Denmark, a fully automated high-throughput phenotyping robot that allows rotating, irrigation, fertilization, weighing, and measuring plants, equipped with thermal and multispectral cameras. In the same light, the National Plant Phenotyping Infrastructure NaPPI in Finland is another example of an automated platform. Such infrastructure enables studying a large number of plants for various agronomic traits. In the category of systems using XY motion is Phenovator, a system for measuring the photosynthesis, growth, and multispectral reflectance of small plants such as Arabidopsis [29]. A more advanced system is presented by PSI (Photon Systems Instruments, Brno, Czechia), named PlantScreenTM XYZ, which works with small and mid-size plants. Such platforms acquire RGB, kinetic chlorophyll fluorescence, hyperspectral, and thermal data through XYZ motion of a robotic arm.

Platforms that acquire images in closed stations require complex conveyor systems, translated into great investments in facilities, hardware, and software. On the other hand, platforms that acquire images in place usually have relatively low efficiency in data acquisition. Another limitation is the requirement of specialized knowledge to control and monitor the systems. Several custom-made affordable systems have recently emerged, broadly categorized based on the type of plant species to study. For instance, “Phenotiki” [30] is an affordable system that analyzes the growth, color, and leaf area of Arabidopsis plants based on a Raspberry Pi single-board computer. Another example is Phenoscope [31], a platform that provides watering and zenithal imaging to monitor plant size and expansion rate during the vegetative stage. An image processing pipeline was also developed to analyze rosette area modeling [32]. These approaches have been developed for rosette-shaped plants, especially Arabidopsis. Other solutions of affordable systems applied on different plant sizes are MVS-Pheno [33] and LCP lab [18], which are portable, low-cost platforms for individual plants. Both platforms work with regular RGB cameras, obtaining a multiview image using a rotatory console. The analysis of data differs with the software; MVS-Pheno developed its software while LCP lab works with freely available software.

Image acquisition and processing are pivotal for the right estimation of plant traits. Plants thus need to be segmented successfully from other objects, such as the background [4,34,35]. However, many of the pipelines available are developed for a narrow range of plant species and, in some cases, require manual work. Solutions are thus needed to enable the phenotypic evaluation of different species under highly controlled conditions, systems that are cost-effective, automated, and that can improve the intensity and accuracy of germplasm selection [3].

In this work, we propose an automated plant phenotyping system called Phenocave built for highly controlled plant growth conditions and regular greenhouses. Instructions to build a similar system and image analysis protocols are provided. The results obtained from the evaluation of Phenocave on wheat (*Triticum aestivum* L.) and sugar beet *(Beta vulgaris* L.) are presented.

## 2. Materials and Methods

### 2.1. Phenocave

**Figure 1:**
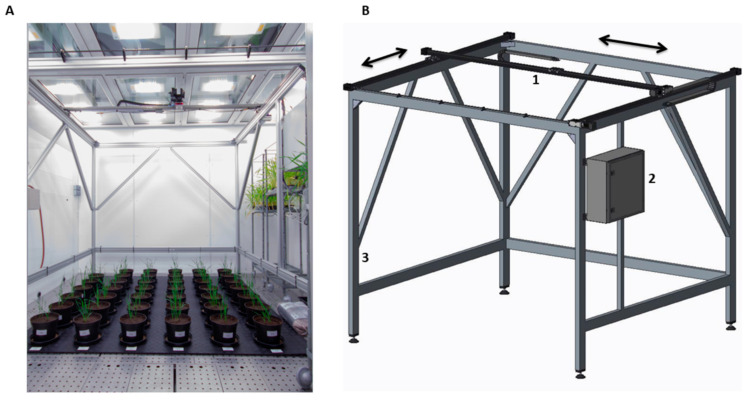
(**A**) Phenocave installed in the Biotron chamber with two imaging sensors mounted, a DSLR Canon EOS and a multispectral MicaSense Altum; (**B**) schematic Phenocave model. The gray arrows indicate the movement on the XY coordinates.

Phenocave has a workspace of 2 × 2 m and 0.5 mm of precision in positioning the camera over the object of interest. It is based on a programmable logic controller (PLC) in conjunction with a programmable motor controller. The system consists of a gantry robot from Igus company (Cologne, Germany) mounted on an aluminum frame structure constructed by Eltech automation (Lomma, Sweden) (Figure 1A). The structural support incorporates two pairs of rigid aluminum legs with reinforcing, attachment points for the gantry, the cabling network with carrier system (Igus, e-chain Black Cable Chain), and an electronic box mounted at the right side of the frame, which contains all the electronic components and the user control system. The gantry robot comprises two linear axis actuators with belt transmission connected to two stepper motors (NEMA23) for XY linear motion.

Engine configuration (camera position, image acquisition speeds, and operating times) is performed with a controller (Drylin Dryve D1). These settings can be configured in the Igus web-based control system for Drylin from any internet browser. All connections and the two motor drivers are connected to a PLC (CPU Siemens S7-1200) that implements a control system over the number of steps (image to acquire) that would be carried out using the XY coordinates and a timer to execute a desirable loop. These parameters can be set up on the display panel (Siemens KTP400 Basic PN) included in the electronic box [36]. To determine the efficiency, reliability, and quality of the obtained results, two case studies were conducted, which included wheat and sugar beet as model plants. The Phenocave parameters were set up based on the position of pots with XY coordinates in mm, the motor speed for image acquisition, and the operating times were fixed for the whole experiment.

### 2.2. Image Acquisition with Phenocave

Individual plant pot images were acquired from top-view using three different imaging sensors attached to the central aluminum plate (Drylin W bearing and mounting plate) from 1.8 m above ground. One RGB digital single-lens reflex (DSLR) camera, a Canon EOS 1300D (Canon U.S.A. Inc., Huntington, NY, USA) with a resolution of 18 megapixels, was mounted with a Canon EF-S 50 mm f/1.8 STM lens. The optimal exposure settings for imaging based on the growth conditions were F-Stop 1/160, exposure time 1/10, AF AI-Servo, and ISO 400. All pictures were saved in 5184 × 3456 pixel JPG format. The second sensor was the MicaSense Altum multispectral camera (MicaSense Inc., Seattle, WA, USA) with an image resolution of 2064 × 1544 pixels and storage in TIF format. The MicaSense Altum has one thermal and five multispectral bands (blue, green, red, red edge, and NIR). As the distance from the top of the plant to the camera lens was less than two meters, it was not possible to align the five multispectral bands perfectly (personal communication with MicaSense support). Thus only the thermal images from the MicaSense Altum were used for analysis. The third sensor was the Canon DSLR Rebel T6 NDVI conversion. Aiming to avoid light reflection and obtain a good background separation from the object of interest (plant leaves) during image processing, a dark floor (interlocking rubber sheets floor) was placed over the original floor of the chamber. Pots were labeled with two identifier numbers according to the position (i.e., row and column). Images were taken every second day to follow the growth conditions (Figure 1B).

### 2.3. Image Acquisition with LCP Lab (Comparative Method)

The results for the wheat case study from Phenocave were compared with a previously published LCP lab system [18]. Pots were individually photographed at every time point from one top-view angle and four side views using two DSLR cameras, Canon EOS 1300D and the 18–55 mm kit lens. Both cameras were tethered to the software digiCamControl [37] with slightly different settings (side view: focal length of 35  mm, F-Stop f/9, and top view: focal length of 30  mm, F-Stop f/10, and both with ISO 400 and exposure time of 1/160 s). The side-view camera was mounted on a tripod 1.5 m away from the target (i.e., plant pots), whereas the top-view camera was maintained at the height of 1 m above the plants. Pots were placed manually on a top-quality Intelligent 360 Photography turntable platform (Shenzhen Comxim Technology Co., Ltd., Shenzhen, Guangdong, China).

### 2.4. Image Processing

#### 2.4.1. Color Images

Green leaf area projection of the plant color images obtained from the Phenocave and the rotatory system was extracted using an image-processing algorithm developed and written in Java as a plugin for ImageJ software (National Institutes of Health, Bethesda, MD, USA). First, a region of interest ROI (plant pot) was selected for each image, and everything outside of it was converted to zero value pixels (black). This was done to minimize the overlapping and non-plant objects in the images. Subsequently, the image was split into red, green, and blue bands to apply a color difference mask obtained by subtracting one color channel from another [18]. For this case, two masks were created, one by subtracting the red channel from the green channel (green minus red), which contrasted most of the plant from other objects. The other mask was obtained by subtracting the green channel from the blue channel (blue minus green) for isolating the leaves missing in the first mask. In both masks and for all time points, a fixed threshold was applied considering that the illumination was always the same and even with values above the pixels belonging to the plant (threshold values (30, 255) and (3, 255)) and discarding the rest of the pixels. This threshold was selected using the option threshold by default of ImageJ and manually adjusted until it reached the desired result. Subsequently, a median filter from the ImageJ toolbox “Remove Outliers” was applied, a filter that replaces a pixel if the median of the surrounding pixels deviates from the median by more than one threshold value (radius = 10 threshold = 50 which = Bright). Finally, both created masks were joined by OR logical operator to be used as a mask to the original image. The resulting image consisted of data of green and yellow leaf pixels and, in some cases, soil. Therefore, a K-means classifier was applied. However, the results were not satisfactory, especially for wheat plants in the maturity stage. Thus, to segment these areas further, a Bayesian classifier was implemented. This machine-learning approach works with a training set of samples previously labeled (green leaves and yellow leaves) which will be the different classes to classify. From them, it creates probability density functions per class, and this way determines the belonging of each new pixel to the class previously defined.

**Figure 2:**
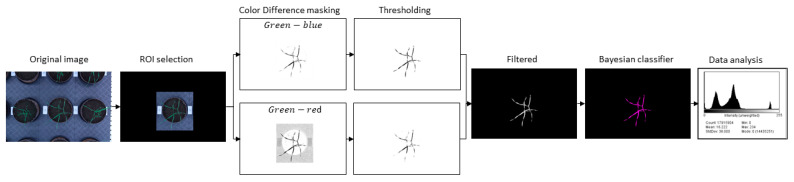
Work flow of image analysis pipeline performed for the extraction of the projected plant biomass from top-view digital images.

The resulting image is grayscale with pixel values ‘0’ (black) for the background regions, ‘1’ (gray) for the green leaves, and ‘2’ (white) for the yellow leaves. The number of pixels of the green leaves was extracted from the histogram. A work flow is presented in Figure 2. For the images of the rotatory method, the scale was selected for every image (one top view and four side views) by using the reference calibration (ruler 300 mm) and calculating pixels/mm, to give a total green area of the plant. The results of the five images were summed.

#### 2.4.2. Thermal Images

**Figure 3:**
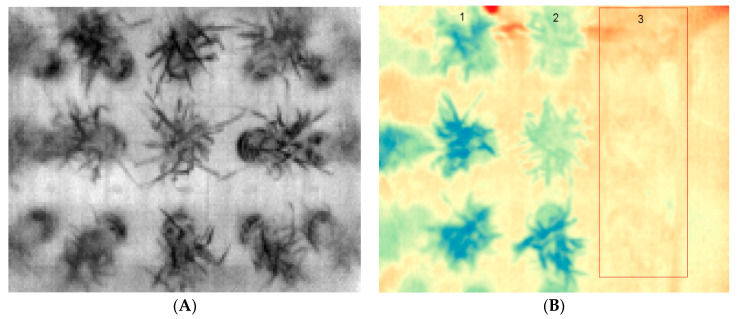
Visualization of a thermal image using the software QGIS (QGIS Geographic Information System): (**A**) left side original image: (**B**) image with pseudocolor, plants in the first and second column (1, 2) show lower near infrared values (color blue), while plants in the third column (3) (red and yellow color) marked with the red frame show higher values (plants under drought conditions).

Thermal images were processed using the free and open-source software QGIS (QGIS Geographic Information System) [38]. First, a set of images was selected after five days of inducing drought, one at stem elongation and one at the heading stage, and another one after two days of normal irrigation for all the plants. The processing steps included the changing of the band rendering, from single-band gray to single-band pseudo color with discrete values from 29,200 (18.85 °C) representing cold (blue) to 29,350 (20.35 °C) hot (red). Afterward, the plant regions to analyze were selected using the option of a circle shape in the polygon tool of the QGIS toolbox, then applying zonal statistics to each shape feature to obtain the mean thermal value for each plant (Figure 3).

### 2.5. Case Studies

Phenocave was evaluated using three different case studies on wheat and sugar beet.

#### 2.5.1. Case Study: Wheat

The experiment was conducted in the Biotron chamber at temperature 23 °C/19 °C (day/night), humidity 50%, and 400 μmol m^−2^ s^−1^ of uniform light intensity with LED lights. A total of 123 seeds of a single spring wheat genotype (provided by Lantmännen Lantbruk) were sown in 41 pots of 3 L (three seeds per pot) with 1.7 kg of soil (Exclusive Flower and Plant Soil with Osmocote). Three treatments were set up with four replicates in two different groups. Group 1 consisted of the control set (28 pots, set 1), while group 2 consisted of three subsets with three types of treatments, namely drought stress at stem elongation (4 pots, set 2), drought stress at the heading stage (4 pots, set 3), and high nitrogen at the heading stage (4 pots, set 4). Plants were placed randomly in the 4 sq m area of the Phenocave workspace. All pots were supplied with 24.99 mg N dosage of ammonium nitrate diluted in 100 mL of water (liquid fertilizer NH_4_NO_3_) before drought treatments were induced.

Digital biomass measurements were obtained across the different growth stages, seedling, tillering, stem elongation, booting, heading, and grain filling [39], in the control set (see image processing section). At the end of each growth stage, four pots were randomly selected (total 24 pots), first to acquire images with the comparative rotatory system (see image acquisition section), then for the conventional process of determining biomass. In this process, shoots were cut and immediately weighed using a precision balance (Sauter RE 3012). Thereafter, shoots were wrapped in aluminum foil and oven-dried at 100 °C, for a period of 24 h. Finally, the dry matter was weighed using the same balance. 

Drought stress conditions in sets 2 and 3 were induced by stopping the irrigation in eight pots at two different time points, four pots at the stem elongation stage (D_SE), 38 days after sowing (set 2), and the other four at the beginning of the heading stage, 45 days after sowing (set 3). Finally, plants were re-watered in both cases after six days of drought, days 44 and 51 after sowing, respectively. At the heading stage, another four pots (set 4) were supplied with 24.99 mg N dosage of ammonium nitrate diluted in 100 mL of water (liquid fertilizer NH_4_NO_3_). The remaining five pots from the control set were allowed to grow until the maturity stage for grain yield determinations. Pots (not under drought treatment) were irrigated every second day with 500 mL of water until the tillering stage, and thereafter the irrigation was increased to 1000 mL. Imaging was continued every second day until the end of the maturity stage (grain filling). Finally, the conventional measurement of biomass was performed for all remaining plants.

- Phenotyping with Handheld Sensors for Wheat

Handheld sensor phenotyping was done from the stem elongation stage (28 plants) until the maturity stage (12 plants), by sampling three times per week and taking three measurements per plant. NDVI measurements were taken with a PlantPen NDVI-300 (Photon Systems Instruments PSI, Drásov, Czech Republic) using three leaves randomly selected from each plant. Then, chlorophyll concentration measurements were taken on the same leaves with an MC-100 chlorophyll concentration meter (Apogee Instruments, Inc., North Logan, UT, USA). Thereafter, QY (PSII Maximum Quantum Efficiency, (Fv/Fm) measurements were taken with a FluorPen FP 100-MAX (Photon Systems Instruments PSI, Drásov, Czech Republic) with detachable blade clips. On each one of the three leaves of the plant, a clip was placed, creating a dark adaptation for 15 min before measurement.

Plant height and leaf area were other parameters measured, but only in the final maturity stage of treatment of plant sets (12 plants). Plant height was measured manually with a ruler from the surface of the soil to the tip of the plant spike. The leaf area was measured in the flag leaf using an LI-3000C Portable Leaf Area Meter (LI-COR Biosciences, Inc., Lincoln, NE, USA). In both cases, three measurements were performed for each pot, and the average of them was considered the final value.

- Analysis of Grain Protein Concentration and Composition

Similarly, as in previous studies [40], the grain protein concentration was evaluated through nitrogen combustion using a nitrogen/carbon analyzer (Flash 2000NC Analyzer, Thermo Scientific, Waltham, MA, USA). The total protein content was calculated by multiplying the total nitrogen content by a conversion factor of 5.7 [41].

Quality of the wheat grain was evaluated following previously described methods [42], determining %UPP (percentage of SDS-unextractable polymeric protein in total polymeric protein) correlating with gluten strength, and TOTE (total SDS-extractable protein) correlating with grain protein concentration [43,44]. Thus, the amount and size distribution of polymeric and monomeric proteins were determined by size-exclusion high-performance liquid chromatography (SE-HPLC) in a two-step extraction procedure [45] with modifications by Johansson et al. [46], extracting SDS-extractable proteins in the first step, and SDS-unextractable proteins by sonication in the second step. SE-HPLC analyses were carried out with the Waters HPLC system (Milford, NH, USA) with a Phenomenex BIOSEP SEC-4000 column (Torrance, CA, USA). The area under the chromatogram was used to calculate %UPP and TOTE following previous methodology [47,48,49,50]. Samples were extracted and run in triplicates.

#### 2.5.2. Case Study: Sugar Beet

The trial was conducted using seeds of a single sugar beet genotype (material provided by MariboHilleshög) exposed to different treatments. As a control, one of the sets consisted of completely untreated seeds (A), while a second set were naked seeds that had undergone a priming procedure (B) to accelerate germination. The proprietary priming process was developed at MariboHilleshög with the specific purpose of enhancing germination speed and early seedling growth. The third set of seeds was pelleted and coated (C), which is common practice for commercially sold sugar beet seeds in most markets. The pellets improve seed drilling performance while the coating contains fungicides and insecticides. The last set of seeds were primed, pelleted, and coated (D) with the purpose to have all the treatments and benefits in one. 

The experiment was organized in a randomized complete block design with four treatments and three replicates. Thirty-six seeds of each treatment (144 seeds) were sown in plastic pots (5 × 5 and 10 cm high) filled with standard garden soil. The 144 plants were evenly distributed per treatment in 12 trays (3 trays per treatment). Then, trays were placed in the Phenocave workspace (4 sq m) in four columns and three rows. Thereafter, plants were allowed to grow in Biotron at 22 °C/18 °C (day/night) temperature, humidity 50%, 350 μmol m^−2^ s^−1^ of uniform light intensity with LED light conditions. 

### 2.6. Statistical Analysis

Statistical analyses were conducted using R [51], analyses of Pearson’s correlations were performed with the conventional biomass measurements and the results obtained from Phenocave. Analysis of variance (ANOVA) was performed with post hoc Tukey’s honest significant difference (HSD) test (*p*-value < 0.05), to evaluate the different effects of treatments and traits measured. For HPLC analysis, Duncan’s multiple range test (DMRT) was performed to estimate specific differences between treatment means.

## 3. Results

### 3.1. Phenocave

The Phenocave automated phenotyping platform comprises an aluminum gantry that can be mounted with various optical sensors as required. In addition, depending on the number of targets, the platform acquires top-view images sequentially with a speed of 0.5–1.5 m/s. Furthermore, it is possible to set up different positions on the XY coordinate with a precision of 0.5 mm in a single cycle per line. A total of 25 pots of 3 L equally distributed in the 2 × 2 m workspace, can take 10 min for imaging and can be repeated in a loop. It is also possible that the camera moves continuously on the *x*-axis, which can be achieved by selecting the home position and a target (used for hyperspectral imaging). The images are stored in the camera for further analysis.

To evaluate Phenocave, two case studies were undertaken using spring wheat and sugar beet.

#### 3.1.1. Case Study: Spring Wheat

##### Group 1

- Image Analysis Results

**Figure 4:**
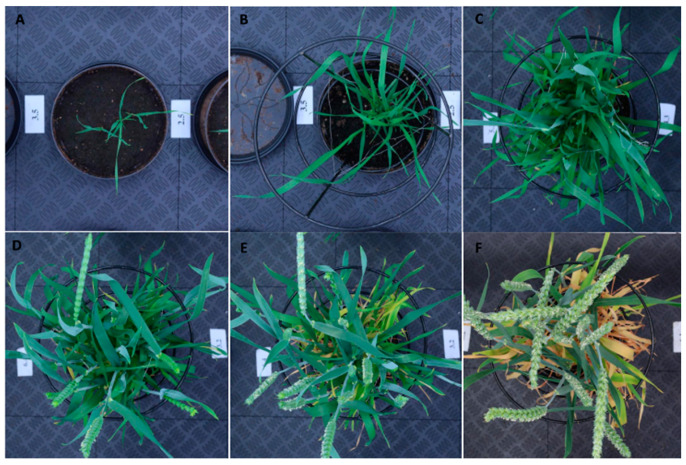
Representative red–green–blue (RGB) images from Phenocave before processing. Spring wheat plants at various growth stages: (**A**) seedling, (**B**) tillering, (**C**) stem elongation, (**D**) booting, (**E**) heading, and (**F**) grain filling.

**Figure 5:**
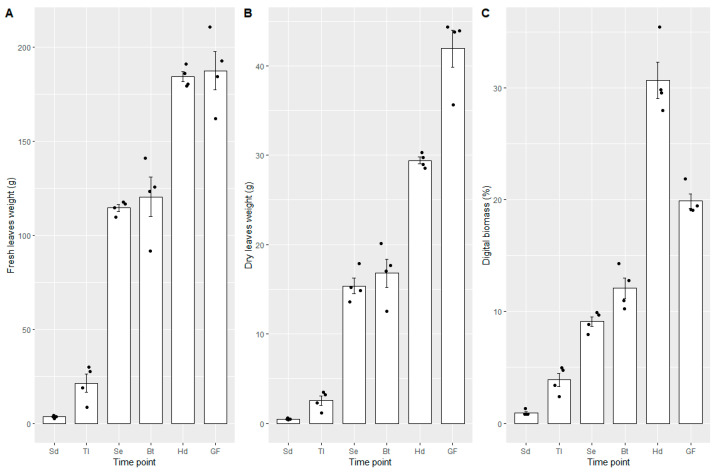
Fresh leaves (**A**), dry leaves (**B**), and digital biomass (**C**) obtained from different growth stages, namely, seedling (Sd), tillering (TI), stem elongation (Se), booting (Bt), heading (Hd), and grain filling (GF). Error bars are standard error.

The image acquisition started when plants reached the seedling stage (seven days post-sowing) and continued three times per week until the end of the grain-filling stage (58 days post-sowing), i.e., during six growth stages, for a total of 360 images. Image segmentation was performed to separate the background and foreground (plant) pixels and segment yellow and green plant material. The RGB images of the plants representing the six time points are presented in Figure 4 and the biomass measurements from digital and conventional methods are presented in Figure 5. There was a progressive development of plants in the six growth stages. The grain filling growth stage had the highest fresh and dry weight values. Whereas for the digital biomass measurements, the highest canopy green cover was at the heading growth stage and it decreased in the grain filling stage.

The digital biomass obtained during the six growth stages was correlated with the conventional biomass measurements results (weight of fresh and dry matter), giving a significant correlation (r = 0.96, *p* < 0.01, r^2^ = 0.92 for dry weight; and r = 0.97, *p* < 0.01, r^2^ = 0.94, for fresh weight). The results of the rotatory system were correlated with the conventional biomass measurements (weight of fresh and dry matter), giving as result a high correlation (r = 0.78, *p* < 0.01, r^2^ = 0.61 for dry weight; and r = 0.88, *p* < 0.01, r^2^ = 0.79, for fresh weight, Table 1).

Furthermore, to estimate time efficiency over conventional biomass measurements and a semi-automatic lab LCP method [18], working time was recorded for wheat plants at the heading stage. The time for conventional biomass measurements took 13 h, distributed in shoot cutting, weighing, oven drying, and weighing. Time with the LCP lab for placing each pot on the platform took around 40 s, setting up the software to acquire images manually and save images took 60 s, finally acquiring a single image took 10 s, thus totaling 2 min per pot. Using the Phenocave system took 15 s to set the number of images and about 15 s to take one image, a total of 0.5 min per image/pot.

##### Group 2

- Image Analysis Results

**Figure 6:**
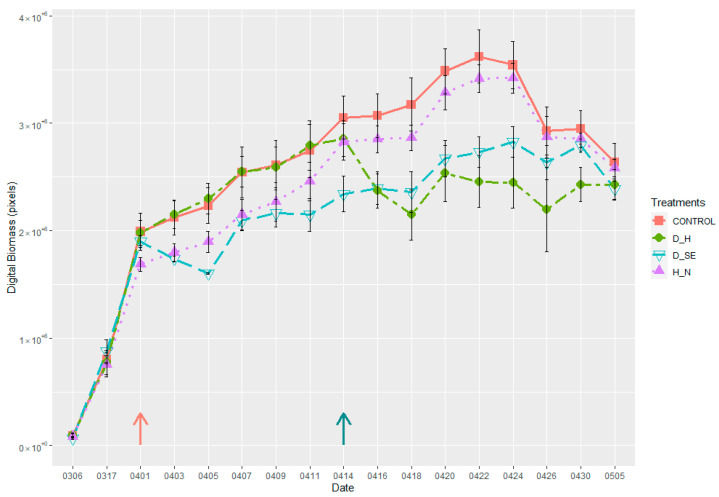
Plant growth pattern under different treatments, (control, drought at stem elongation (D_SE), drought at heading (D_H), and high nitrogen content (H_N)), from sowing to grain development. Pink arrow indicates when drought stress was induced, D_SE; dark cyan arrow indicates when drought stress was induced, D_H, and second nitrogen dosage was applied, H_N. The *Y*-axis represents the time points when the digital biomass was sampled and the *X*-axis represents the mean values of the obtained digital biomass. Error bars are standard error.

**Figure 7:**
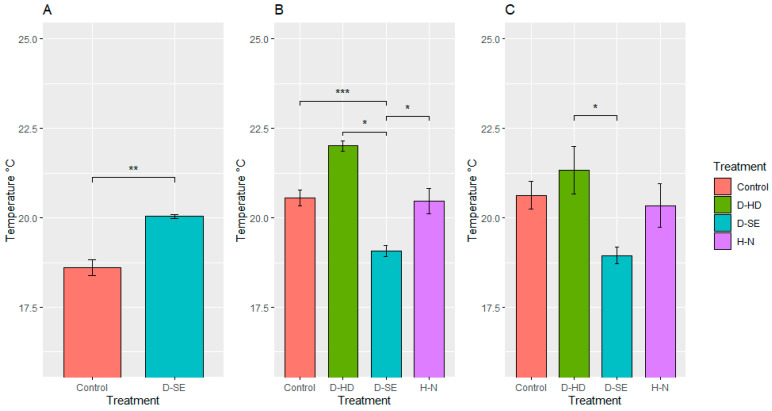
Surface temperature of plants under different treatments, (control, drought at stem elongation (D-SE), drought at heading (D-HD), and high nitrogen content (H-N)). The *X*-axis represents the treatments and the *Y*-axis represents the mean temperature values obtained with QGIS software. Statistically significant differences (Tukey’s HSD *** *p* < 0.001, ** *p* < 0.01 and * *p* < 0.05) are denoted by the stars above the bars. Error bars are standard error. Temperature measurements (**A**) after five days of inducing drought stress at stem elongation, D_SE and (**B**) after five days of inducing drought stress at heading, D_HD, and second dosage of liquid nitrogen fertilizer was applied, H-N. (**C**) Temperature measurements when all plants were irrigated.

There was a strong impact of the induced treatments on plant growth rates. As shown in Figure 6, plants under control conditions grew well, showing a high level of green canopy biomass compared to the plants under stress. Significant improvements were observed in plant performance after the second application of nitrogen fertilizer at the heading stage (marked with a dark cyan arrow). Plants under drought treatment showed lower growth once drought treatment was induced (marked with rose and dark cyan arrows). Even though irrigation was withheld for the same amount of time in the two drought treatment sets, drought impacted differently during the two growth stages. There was a greater reduction in the plant biomass during the drought at the heading stage compared to the stem elongation stage. Significant differences in canopy cover were observed in the drought-stressed and control plants also based on thermal imaging (Figure 7). The differences were reduced once the plants were allowed to recover from stress. Likewise, significant differences (*p* < 0.05) were found in the digital biomass among plants under different treatments four days after the treatments were induced (Table 2).

- Handheld Sensor Measurements

**Figure 8:**
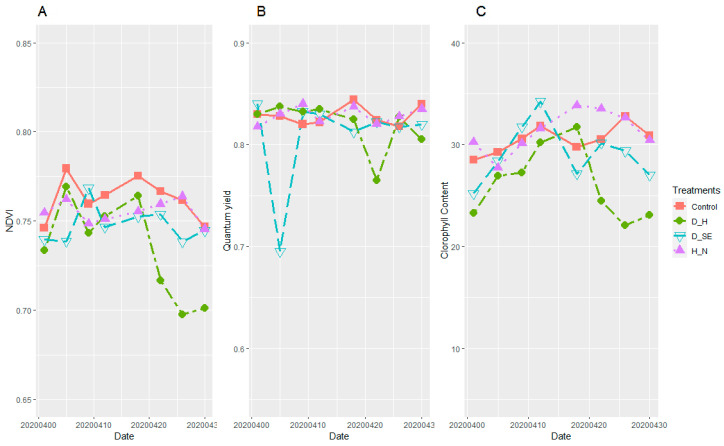
Plants development curves obtained with handheld sensors versus sampling dates (from stem elongation to grain filling stage), clustered based on their treatment forming four groups (control, drought at stem elongation (D_SE), drought at heading (D_H), and high nitrogen content (H_N). (**A**) Normalized difference vegetation index (NDVI); (**B**) quantum yield (QY); (**C**) chlorophyll content index (CC).

The parameters measured with the handheld sensors—NDVI, chlorophyll content (CC), and quantum yield (QY) showed statistically significant differences among the different treatments along the measured time points. Plants showed normal values (NDVI = 0.75, CC = 30, QY = 0.84) during the first two growth stages (seedling and tillering), then values increased (NDVI = 0.78, CC = 35, QY = 0.84) in the next two growth stages (stem elongation and booting); in the last two time points (heading and grain filling), values decreased again to the initial values. In the case of plants exposed to drought at stem elongation, values were lower (NDVI = 0.73, CC = 27, QY = 0.69). Plants set under drought at heading also showed lower values (NDVI = 0.68, CC = 20, QY = 0.75), while for the plants with high nitrogen content values increased (NDVI = 0.72, CC =34, QY = 0.82) (Figure 8).

- Protein Content and Quality

As described in the methods, grain protein concentration was measured using combustion, while grain protein quality was obtained from TOTE and %UPP values measured with SE-HPLC. The grain protein concentration in the wheat was generally low (Table 3), ranging from 7.7% to 13%. The highest grain protein concentration was found in samples that were drought treated at heading, with a mean protein concentration of 11.7%. The control samples and those subjected to drought under stem elongation showed the lowest grain protein concentrations of 8.4% and 8.0%, respectively, and did not differ significantly. The high nitrogen treatment resulted in samples differing from those of the other treatments and with a mean grain protein concentration of 9.4% (Table 3). The grain protein concentration measured through combustion correlated significantly (*p* < 0.01) with TOTE values obtained from the SE-HPLC. Thus, similarly as for grain protein concentration, the highest TOTE values were found for samples subjected to drought at heading, followed by samples subjected to high nitrogen treatment, and significantly lower values were seen for the control samples and the samples subjected to drought at stem elongation (Table 3). Significantly, the highest %UPP values, indicating the highest gluten strength among samples, were obtained for those samples subjected to drought stress at heading, while for the other treatments, no significant differences were revealed for %UPP (Table 3).

The clearest correlation (*p* < 0.005 for mean values) between grain protein concentration and any of the above described Phenocave parameters was (positive) for the surface temperature at watering after drought treatment (Figure 7C). Drought at heading, which was the treatment mainly affecting the grain quality (Table 3, %UPP), was also the treatment that most clearly showed an effect on several of the Phenocave parameters, including plant growth (Figure 6), surface temperature after drought treatment at regular watering (Figure 7C), NDVI (Figure 8A), and chlorophyll content index (Figure 8C).

#### 3.1.2. Case Study: Sugar Beet

**Figure 9:**
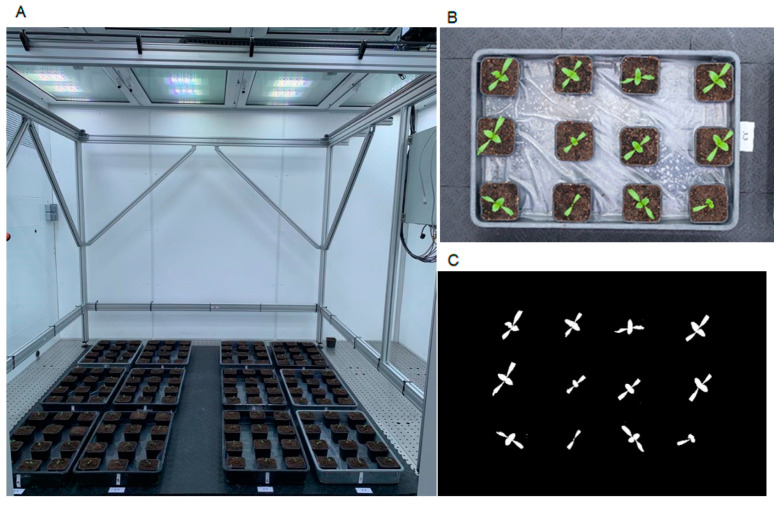
(**A**) Sugar beet experiment installed in Phenocave; (**B**) individual image of sugar beet plants per tray; (**C**) image after processing with the developed pipeline.

**Figure 10:**
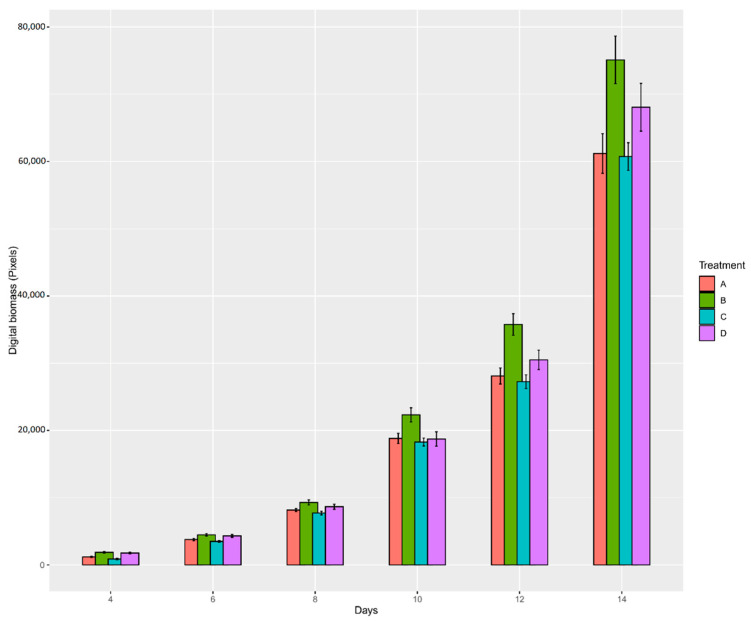
Plant germination and development of plants from seeds with different treatments: (**A**) control; (**B**) primed; (**C**) pelleted and coated; and (**D**) primed, pelleted, and coated; from sowing to 15 days after sowing. The *Y*-axis represents the digital biomass in pixels and the *X*-axis represents sampling days.

The projected leaf area trait in the 12 trays was quantified three times a week during the early growth stage (seedling) for 18 days (Figure 9). During this time, a different germination speed was observed among the sugar beet treatment sets (Figure 10). Two of the treatments, B (primed) and D (pelleted, primed, and coated), had the fastest germination, which could be observed in the majority of samples after four days of sowing. However, the seeds that were not exposed to priming treatment, namely treatment A (control) and C (pelleted and coated), showed a delay in germination, where only half of the samples showed germination after four days. Nonetheless, the germination was 100% in all the treatment sets ten days post-sowing. 

Statistically significant differences in plant development by canopy measurement were obtained in four time points of measurement, 5, 8, 12, and 15 days after sowing for all sets of treatment, *p* < 0.01 ** and *p* < 0.05 * in the first and second time points, respectively (Table 4). The differences decreased in the next two time points, *p* < 0.05 *, between treatments B and C. There were no significant differences in the next time points, 18, 21, and 24 days after sowing, when plants grew under optimal conditions.

## 4. Discussion

Phenocave is a platform highly automated for plant phenotyping, and once the program is set up, it requires minimal human intervention. Image acquisition for different types of plants is one of the issues presented in some low-cost phenotyping platforms [30,31,34,52], while Phenocave can be used with any type of plant size. In addition, the acquisition efficiency of the platform is another advantage over other similar platforms. Approximately 10 min are required to acquire a set of 25 big plant images using Phenocave and they can be analyzed in less than 15 min, whereas the semi-automated LCP lab [18] requires 30 min and labor to implement one of the suggested free software packages. The Phenocave system is user-friendly, which facilitates its application for researchers with different scientific expertise. Furthermore, Phenocave can be set up in a controlled growth environment. Nonetheless, a resulting limitation of Phenocave is that the workspace is only 2 × 2 m, allowing the assessment of up to 25 large plant pots of 3 to 5 L and about 150 small ones of less than 2 L. Despite the fact that the workspace makes it difficult to handle more than 30 big plants, the pots remain static without causing any possible mechanical damage to the plant leaves or any secondary effect on the expression of the phenotype. Another advantage of this setup is that plants can be analyzed by different sensors in the same run, reducing the amount of time between data acquisition for the same individual.

Experiments carried out under controlled environments are often difficult to associate in terms of yield performance under field conditions. The flexibility of specific environmental conditions and the control of the exposure of the plants by modifying those conditions is one of the biggest advantages of growing plants under controlled environmental conditions [53]. Many experiments in indoor controlled environments evaluate different parameters of plant growth under different conditions. However, many of them imply high costs, a lot of labor, specialized knowledge, and not all of them work for all types of plants. Thus, to improve the speed, accuracy, costs, and reliability of this process Phenocave was developed as an automated phenotyping system to evaluate visual traits from top-view plant images. Phenocave provides easy accessibility, especially because of its good cost-performance ratio; moreover, the pipeline developed is in ImageJ, an open-source software, and its architecture is flexible, which allows it to be unmounted and mounted in other indoor environments, and the interface is user-friendly.

Positive correlations were observed between projected green area obtained from imaging and destructively harvested green and yellow leaf biomass in all the growth stages (Table 1). The correlation had a slight decrease at the stem elongation stage because of the number of overlapping leaves (Figure 4C). Plants with overlapping leaves evaluated with image analysis can cause underestimation of the projected area [54,55]. Despite this correlation reduction, the results generally were highly correlated. Studies in controlled greenhouse conditions have found similar correlations between areas estimated by image analysis and harvested biomass [4,35,54,55] which indicates the applicability of top imaging with Phenocave for non-destructive evaluation of germplasm.

Understanding the impact of drought at different growth stages contributes to the efficiency of breeding drought-tolerant wheat varieties. Important factors such as the intensity and frequency of drought affect the performance of any crop. The plant developmental stage at which drought events occur is equally important [56]. While testing Phenocave to identify the response of plants to drought at two growth stages, we found negative effects in wheat, such as reduction of leaf biomass production and grain yield. Nonetheless, the duration, intensity, and timing of certain stresses differ in how and which yield components are affected [57,58,59]. In the present study, the stress was imposed over six days of stopping the irrigation, resulting in visual differences in shoot biomass in the plants (Figure 6). The occurrence of drought stress at the stem elongation stage led to a significant reduction in leaf biomass and fewer spikes. Previous studies have shown that drought stress at the stem elongation stage greatly decreased the grain yield compared to booting and grain filling stages [60]. Results of drought stress at the heading stage showed a significant decrease in the number of grains per head, grain weight, and leaf biomass which was even more severe than in the early stage.

As for the wheat case study, the HPLC analysis revealed a low grain protein yield (7.7–13%) compared to field-grown wheat (normally 11–14% in Swedish field conditions), which indicates the need to fertilize wheat to a level around the high nitrogen treatment conditions used in the present experiment. Despite the generally low grain protein concentrations obtained here, the present results corresponded with those previously reported [61,62]; for example, that reduced green biomass is a major effector on grain protein concentration. Thus, a drought at heading resulted in a severe decrease of plant biomass and thereby a reduction of carbohydrate transportation to the grain, which resulted in a high grain protein concentration. The negative correlation between TOTE and %UPP reported in several studies [43,44,50] was not seen for samples with different treatments in the present study. The relatively high gluten strength, verified by correlating %UPP values [43,44], found in the samples subjected to drought at heading, is most likely due to an increase in hydrogen and disulfide bond formation, previously reported as an outcome of decreased precipitation and increased temperature [48,61,62].

The second case study was the effect of sugar beet seed treatments on germination and plant growth. The results indicated that seeds under priming treatment were positively influenced in terms of germination ability and speed, especially during the first four days. This is the effect of priming seeds, which causes an acceleration of germination and the acceleration of seedling growth [63]. On the other hand, pelleting and coating treatment reduced germination rates, especially in the early stages. The delayed seed germination can be explained by the slow water flow from the germination medium through the pellet and pericarp to the seed [64]. Hence, although necessary from an agricultural perspective, pelleting and coating may slightly delay germination, while priming may compensate for this delay. Previous research showed that primed seeds with a lower level of vigor showed a faster and higher germination ability than non-primed seeds with higher vigor [64]. Despite this effect caused by both treatments, after 14 days of germination, the differences in seed germination and plant growth development diminished.

An advantage of using Phenocave is the free choice of the use of the imaging sensors supported by Phenocave (RGB, multispectral, NDVI, multispectral) and the space (2 sq m) within which plants can be placed anywhere in the imaging region as required by the experimental design. While the current setup of Phenocave is limited to manual data transfer and manual irrigation, this might be included in the automatic system in future modifications. Multispectral bands from the Micasense Altum sensor were not available in Phenocave since the short distance between the lens and the object was problematic for band alignment. Several algorithms have been developed to identify keypoint feature descriptors for band alignment [65]. Keypoint descriptors assist in better alignment when the alignment bands have nearly uniform reflectance profiles and unique patterns in the scene [66]. Thus, in future work, an automated work flow will be developed for the alignment of bands from the Micasense Altum camera for pictures taken in close range. An additional improvement could be regular radiometric calibration of the thermal sensor of the Micasense Altum camera. A previous study showed that radiometric calibration of thermal sensors produces more accurate results [67]. Thus, a similar calibration of sensors could be beneficial for experiments requiring higher measurement accuracy. Nonetheless, this study demonstrates that the Phenocave system is a useful tool for the non-destructive estimation of plant shoot biomass under different growth conditions and at various time points. 

## 5. Conclusions

In order to adapt to climate change, unexpected agronomical diseases, and other factors that affect plant performance, it is of utmost importance to assess plants under different environmental conditions in a fast, accurate, friendly, and affordable manner. Phenocave offers the opportunity to assess the visual traits of the plants under highly controlled environments. In addition, the platform reduces manual work for the users because of a high level of automation. It moves in XY direction acquiring high-quality individual photographs of plants with different imaging sensors (RGB, thermal, NDVI, and hyperspectral), which allows the extraction of different characteristics of plants. 

One of the advantages of Phenocave is that it evaluates small- to mid-size plants, such as sugar beet and small-grain cereals. Besides, it can be unmounted and mounted to be used in other environments. This study highlights the potential of developing systems similar to Phenocave which promote work time efficiency, cost efficiency, and flexibility. In future work, data transfer and management will be further improved, and broader applications such as plant diseases and automatic irrigation will be studied, which can further contribute to allowing faster evaluation of plant responses to various treatments. 

## Figures and Tables

**Table 1:** Pearson correlation coefficient between digital biomass, fresh weight, and dry weight at different growth stages.

Wheat Growth Stage According to Zadok Scale	Phenocave		Rotatory System	
	Digital Biomass (Pixels) vs. Fresh Leaves (g)	Digital Biomass (Pixels) vs. Dry Leaves (g)	Digital Biomass (Pixels) Fresh vs. Leaves (g)	Digital Biomass (Pixels) vs. Dry Leaves (g)
Seedling	0.82	0.82	0.95	0.93
Tillering	0.99	0.99	0.92	0.91
Stem Elongation	0.52	0.84	0.67	0.62
Booting	0.90	0.87	0.96	0.97
Heading	0.89	0.85	0.85	0.57
Grain Filling	0.89	0.62	0.95	0.67

**Table 2:** Differences among the four treated plant groups after treatment effect: drought at stem elongation (D_SE), drought at heading (D_H), and high nitrogen (H_N). Significant differences marked with asterisk (* *p* < 0.05, ** *p* < 0.01).

Treatment		Pr(>|t|) Significant Value of Projected Green Area after Treatment	
		Treatment D_SE	Treatments D_H and H_N
Control	D_H	0.71	0.001 **
Control	D_SE	0.003 **	0.008 **
D_H	D_SE	0.002 **	0.37
Control	H_N	0.07	0.483
D_H	H_N	0.04 *	0.006 **
D_SE	H_N	0.14	0.04 *

**Table 3:** Mean values of grain protein concentration, as well as of total SDS-extractable proteins (TOTE) and percentage of SDS-unextractable polymeric protein in total polymeric protein (%UPP) obtained from size exclusion-high performance liquid chromatography for wheat subjected to different treatments; control, drought at heading (DH), drought at stem elongation (DSE), and with high nitrogen application (HN). Numbers within the same column followed by the same letter do not differ significantly at *p* < 0.05 by Duncan post hoc test.

Treatment	Grain Protein Concentration (%)	TOTE (10^7^)	%UPP
CONTROL	8.4 c	6.72 c	64.5 b
DH	11.7 a	9.05 a	70.7 a
DSE	8.0 c	6.46 c	61.7 b
HN	9.4 b	7.75 b	60.3 b

**Table 4:** *p*-Value analysis of the plant germination rate of plants’ seeds with different treatments: (**A**) control (intercept); (**B**) primed; (**C**) pelleted and coated; and (**D**) primed, pelleted, and coated; during the different days sampled. Significant differences marked with asterisk (* *p* < 0.05, ** *p* < 0.01 and *** *p* < 0.001).

Treatment	Pr(>|t|) Significant Value of Germination after Sampling					
	1 Day	3 Days	5 Days	7 Days	9 Days	10 Days
(Intercept)	1.01 × $10^{-5}$ ***	1.94 × $10^{-8}$ ***	2.97 × $10^{-8}$ ***	1.26 × $10^{-7}$ ***	1.17 × $10^{-7}$ ***	1.39 × $10^{-6}$ ***
B	0.003 **	0.02 *	0.04 *	0.0567	0.01 *	0.11
C	0.12	0.21	0.61	0.724	0.50	0.87
D	0.009 **	0.06.	0.13	0.4474	0.24	0.35
